# Protocol optimization for simultaneous DNA and RNA co-extraction from single hard tick specimens

**DOI:** 10.1016/j.mex.2021.101315

**Published:** 2021-03-21

**Authors:** Alessandra Cafiso, Giulia Chiappa, Camilla Luzzago, Anita Koni, Daniele Bonato, Xhelil Koleci, Chiara Bazzocchi

**Affiliations:** a Department of Veterinary Medicine, Università degli Studi di Milano, Lodi, Italy; b Coordinated Research Center "EpiSoMI", Università degli Studi di Milano, Milano, Italy; c Department of Veterinary Public Health, Faculty of Veterinary Medicine, Agricultural University of Tirana, Tirana, Albania; d Pediatric Clinical Research Center "Romeo ed Enrica Invernizzi”, Università degli Studi di Milano, Milano, Italy

**Keywords:** Nucleic acids, Purification, DNA isolation, RNA isolation, Co-extraction, Tick, PCR, Reverse transcription, Coinfection, Vector-borne pathogen

## Abstract

Hard ticks are important vectors of DNA- and RNA-based infectious microorganisms, but they also host complex microbial communities in which pathogens and symbionts can interact among each other and with the arthropod host itself. Molecular investigations on ticks and their hosted microorganisms are important for human and animal health. These analyses often imply the use of both DNA and RNA, with prompt preservation of nucleic acids after collection, and safe handling in case of low-level containment. Several commercial kits are available for the co-extraction of DNA and RNA; however, cost can be a limiting factor for the choice of this method, which also require additional reagents for nucleic acids preservation before extraction. Additionally, extraction buffers provided in commercial kits do not guarantee the safe handling in case of hazardous biological material. With the aim of epidemiological screenings for tick-borne pathogens and gene expression analyses focused on the relationship among ticks and their microbial communities, an optimized protocol for DNA and RNA co-extraction from single adult hard tick specimens (Ixodidae) has been developed using TRIzol^Ⓡ^ LS Reagent.A method for

• DNA/RNA co-extraction from adult hard tick specimens;
• Safe sample handling;
• Obtaining DNA and RNA simultaneously for diagnostic procedures and RNA for gene expression/transcriptomic analyses.

## Graphical abstract

**Figure:**
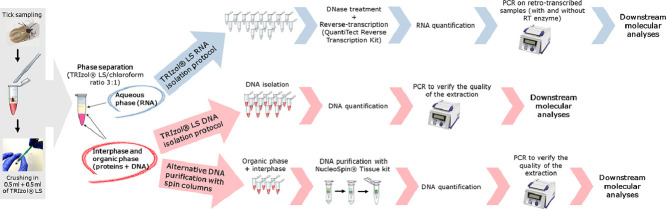

Specifications Table

**Table:** 

Subject Area:	Veterinary science and veterinary medicine
More specific subject area:	Veterinary parasitology, vector-borne pathogen, zoonotic disease, microbial community.
Method name:	DNA/RNA co-extraction from hard tick specimens
Name and reference of original method:	Chomczynski, P. A reagent for the single-step simultaneous isolation of RNA, DNA and proteins from cell and tissue samples. BioTechniques 15 (3) (1993) 532–537 [1].
Resource availability:	https://www.thermofisher.com/order/catalog/product/10296028

## Method details

Here we describe a method for the co-extraction of DNA and RNA form single adult unengorged and semi-engorged tick specimens using TRIzol^Ⓡ^ LS Reagent (Invitrogen, Thermo Fisher Scientific Inc., Waltham, MA, USA; hereafter “TRIzol^Ⓡ^ LS”). This method permits optimal DNA and RNA co-extraction, with the use of a moderately low-cost reagent, allowing a safe handling of the samples, especially when working with low-level containment (biosafety level-2). The current method is suitable for multiple molecular analyses, in particular epidemiological studies aimed at investigating the presence of DNA- and RNA-based tick-borne pathogens (TBPs) in the same sample. Moreover, it is applicable to transcriptomics and gene expression analyses on ticks and their microbial communities, and supports studies aimed at understanding the tick metabolism and biological interactions between ticks and their symbionts, or between ticks and tick-borne pathogens, or both.

### Samples preparation and processing

A total of 11 *Ixodes ricinus* and eight *Hyalomma* spp. adult females were collected in different areas of Albania in 2018. In detail, fourteen semi-engorged specimens (five *I. ricinus* and eight *Hyalomma* spp. individuals) were collected from domestic ruminants, while five unengorged *I. ricinus* females were collected from vegetation. Samples were stored alive in 50 ml conical tubes, transported to a laboratory facility and processed the same day (to avoid nucleic acids degradation) using TRIzol^Ⓡ^ LS under a chemical fume-hood. Single tick individuals were washed in 1X PBS and dried on paper towels, identified under a stereomicroscope [2,3], and immediately processed. The extraction of nucleic acids with TRIzol^Ⓡ^ LS was based on volumes and procedure steps indicated in the manufacturer's protocol. Briefly, each tick specimen was placed in a sterile RNase-/DNase-free 1.5 ml polypropylene tube with 500 µl of TRIzol^Ⓡ^ LS, subsequently cut with a sterile scalpel (blade size 11) and directly crushed with a sterile pestle. Additional 500 µl of TRIzol^Ⓡ^ LS were added to the sample and mixed thoroughly. The TRIzol^Ⓡ^ LS/crushed tick mixtures were subjected to a “stopping point” at -20 °C, as indicated by manufacturer's instructions to allow the samples to be stored frozen for prolonged time. Samples were shipped frozen under controlled temperature to a second laboratory facility for subsequent procedures of nucleic acids isolation and further molecular analyses.

### RNA and DNA co-extraction

After thawing the samples stored in TRIzol^Ⓡ^ LS, phases separation was performed by adding chloroform in a TRIzol^Ⓡ^ LS/chloroform ratio 3:1, based on manufacturer's instructions, under a chemical fume-hood. For each sample, aqueous phase was set apart from the other two (interphase/organic phase) and subjected to RNA isolation according to TRIzol^Ⓡ^ LS protocol [1]. The final resuspension of RNA samples was performed in 40 µl of RNase-free water, followed by an incubation at 56 °C for 10 min for a complete resolubilization of the nucleic acid. RNA samples were immediately stored at -80 °C until subsequent use.

DNA present in the interphase/organic phase was purified based on TRIzol^Ⓡ^ LS manufacturer's instructions (performed on 13 interphase/organic phases obtained respectively from 13 samples). DNA isolation was carried out with a final resuspension in 50 µl of 1X TE buffer (Tris-EDTA: 10 mM Tris, 1 mM EDTA, pH 8.0). Insoluble materials (e.g. sclerotized body parts, such as cuticle) were pelleted by centrifugation for 10 min at 12,000 ×g at 4 °C. The supernatant was transferred to a new 1.5 ml tube and stored at -20 °C until use.

In addition, six interphase/organic phases (obtained respectively from the remaining six samples) were subjected to DNA purification using the commercial kit NucleoSpin^Ⓡ^ Tissue kit (Macherey-Nagel, Düren, Germany) to reduce the required procedure time (about 1.5 h versus 2.5 h circa needed in TRIzol^Ⓡ^ processing) and compare the quality of DNA samples isolated with both protocols. In detail, the interphase/organic phases were treated following TRIzol^Ⓡ^ LS manufacturer's protocol (steps 1a-1f of TRIzol^Ⓡ^ LS Reagent user guide for DNA isolation), with precipitation in 100% ethanol [4]. The precipitated material was resuspended in 180 µl of Buffer T1 (NucleoSpin^Ⓡ^ Tissue kit) and DNA isolation was performed following the kit manufacturer's protocol. DNA elution was performed with 50 µl of 70 °C pre-warmed Buffer BE. Purified DNA samples were stored at -20 °C until subsequent analyses.

The quantification of the extracted DNA and RNA samples was performed using Qubit 3.0 Fluorometer (Qubit RNA Broad-Range Assay Kit and Qubit DNA Broad-Range Assay Kit; Thermo Fisher Scientific, Inc., Carlsbad, CA, USA), the purity was evaluated using a spectrophotometer (Nanodrop ND-1000 Spectrophotometer; Thermo Fisher Scientific, Waltham, MA, USA) and the integrity was determined using Agilent TapeStation 2200 (Agilent, Santa Clara, CA, USA). Information about nucleic acid concentration (ng/µl), and 260/280 and 260/230 ratios values are shown in Table 1. In addition, the integrity of the extracted RNA, which is pivotal for possible subsequent gene expression and/or transcriptomic analyses, is indicated as RNA integrity number (RIN). The median of the RIN values was 8.95, which suggests a good integrity of the RNA, since a RIN of 10 represents highly intact RNA (electropherograms are shown in Supplementary Fig. 1).

**Table 1:** Data obtained on unengorged and semi-engorged tick specimens tested for the protocol optimization after: (i) RNA extraction of 19 samples using TRIzol^Ⓡ^ LS Reagent, (ii) DNA extraction of 13 samples following TRIzol^Ⓡ^ LS Reagent manufacturer's protocol and (ii) DNA extraction of six samples using a commercial kit (NucleoSpin^Ⓡ^ Tissue kit, Macherey-Nagel, Düren, Germany). Data for each extraction method are expressed as nucleic acid concentration per microliter (ng/µl) obtained by Qubit 3.0 instrument, and 260/280 and 260/230 ratios obtained using NanoDrop 1000 spectrophotometer.

ID	Tick species	Tick engorgment phase	RNA (TRIzol^Ⓡ^ LS)			DNA (Trizol^Ⓡ^ LS)			DNA (commercial kit)		
			ng/µl	260/280	260/230	ng/µl	260/280	260/230	ng/µl	260/280	260/230
1	H	SE	517	1.95	0.86	275	1.87	2.08	ND	ND	ND
2	H	SE	297	2.04	1.58	65	1.66	1.41	ND	ND	ND
3	H	SE	203	2.01	1.78	78	1.77	1.53	ND	ND	ND
4	H	SE	104	2.04	1.72	89	1.66	1.72	ND	ND	ND
5	H	SE	672	1.88	1.90	115	1.62	0.87	ND	ND	ND
6	Ir	SE	519	1.85	0.59	230	1.53	0.87	ND	ND	ND
7	Ir	SE	250	2.05	1.68	181	2.13	2.03	ND	ND	ND
8	Ir	SE	552	2.05	1.34	33	2.18	2.74	ND	ND	ND
9	Ir	SE	696	2.00	1.72	56	1.62	0.11	ND	ND	ND
10	Ir	SE	106	2.04	1.98	87	2.18	1.10	ND	ND	ND
11	Ir	U	399	2.03	1.88	285	1.97	1.35	ND	ND	ND
12	Ir	U	1605	2.02	1.56	27	2.13	2.20	ND	ND	ND
13	Ir	U	369	2.04	2.07	218	1.96	2.31	ND	ND	ND
14	Ir	SE	1302	1.89	1.10	ND	ND	ND	158	2.05	2.40
15	H	SE	720	1.97	1.29	ND	ND	ND	25	2.06	2.21
16	H	SE	516	2.03	1.79	ND	ND	ND	81	2.12	2.30
17	H	SE	987	2.01	1.51	ND	ND	ND	154	1.97	2.20
18	Ir	U	258	2.03	1.35	ND	ND	ND	350	2.13	2.31
19	Ir	U	104	2.00	1.62	ND	ND	ND	36	1.97	2.30

Ir = *Ixodes ricinus*, H = *Hyalomma* spp., U = unengorged, SE = semi-engorged, ND = not done.

RNA and DNA yield variabilities were observed, probably due to different tick engorgement phases (Table 1). In fact, nucleic acids belonging to the arthropod, vertebrate host's blood and tick microbial community can affect the total amount of the extracted genetic material. Furthermore, when compared, extraction results obtained by isolating DNA using TRIzol^Ⓡ^ LS protocol and the commercial kit showed similar DNA concentrations (ng/µl) as well as similar 260/280 ratio (*p* > 0.05 for both). However, lower 260/230 ratio values were observed for those samples extracted using TRIzol^Ⓡ^ LS compared to the commercial kit (*p* = 0.027). This difference is probably related to the higher efficiency of the kit-based isolation in removing contaminants, such as phenol, that can decrease 260/230 ratio values. Finally, nucleic acids yield variability did not affect the PCR results for any of the extracted sample (as indicated in ‘PCR validation on DNA and cDNA samples’ section).

### cDNA preparation and purity check

cDNA was synthetized starting from 800 ng of total RNA using the QuantiTect Reverse Transcription Kit (Qiagen, Hilden, Germany), with genomic DNA (gDNA) removal step carried out following manufacturer's instructions. In fact, the presence of contaminant DNA in some of the isolated RNA samples was confirmed through RNA quantification before and after DNase treatment (5–10% of DNA contamination; RNase-Free DNase set, Qiagen, Hilden, Germany). gDNA-removal step is pivotal in case of transcriptomics and gene expression analyses, but can be surely avoided in case of studies aimed at the detection of viral RNA.

Reverse-transcription (RT) step was performed at 42 °C for 20 min and RT Primer Mix (containing random primers and oligo-dT) was used to ensure cDNA synthesis from all regions of RNA. Additional reactions without retrotranscriptase enzyme were performed for each sample to verify the complete DNA removal (RT-control).

### PCR validation on DNA and cDNA samples

To assess the amplifiability of the extracted DNA and the absence of gDNA in RNA samples, a ~650 bp fragment of the tick *cytochrome oxidase I* (*COI*) gene was amplified [5]. PCR reactions were performed on DNA, cDNA and RT-control samples, applying the thermal profile published by the authors with annealing temperature set at 53 °C for 30 s [5]. *I. ricinus* specimens where also tested for the presence of the endosymbiont *Midichloria mitochondrii* using primers for the amplification of a 1100 bp long 16S rDNA gene fragment [6]. PCR reactions were performed on both undiluted and 1:10 diluted samples, to evaluate the eventual impact of inhibitors due to non-optimal 260/280 and 260/230 ratios obtained during spectrophotometric analyses.

Amplicons were loaded on 2% agarose gel. Both *COI* and 16S rDNA protocols applied to DNA samples (performed respectively on all the 19 tick specimens and on the 11 *I. ricinus* individuals) showed the presence of a clear band at the expected molecular size, regardless the starting DNA concentration. Additionally, the amplification of *COI* fragment from cDNA samples showed the effective reverse-transcription and the complete removal of gDNA (since no amplicons were observed for RT-controls).

## Additional information

Ticks are hematophagous arthropods that can harbor a variety of microorganisms, from prokaryotic endosymbionts to infectious viruses, bacteria and protozoa. The simultaneous extraction of DNA and RNA is essential for the study of these organisms, starting from an epidemiological point of view to the biological evaluation of the interaction between ticks and their microbial communities.

The DNA and RNA co-extraction from a single specimen is commonly performed using commercial kits, which have higher costs per sample compared to TRIzol^Ⓡ^ LS approach, and do not assure a prompt and complete inactivation of infectious agents during the first steps of sample handling. For example, transmission of Crimean-Congo Haemorrhagic Fever virus can also occur through handling or squashing of infected ticks [7]. TRIzol^Ⓡ^-based reagents are chemical compounds conceived for the extraction of RNA, DNA and proteins with guanidinium thiocyanate and phenol. TRIzol^Ⓡ^ LS has a slightly more concentrated formula compared to TRIzol^Ⓡ^ Reagent (Invitrogen, Thermo Fisher Scientific Inc., Waltham, MA, USA). Indeed, the use of TRIzol^Ⓡ^ LS has been shown to inactivate members of different infectious virus genera (including members of Bunyavirus and Flavivirus; [8,9]). This could allow a safer manipulation of the samples when working with low-level containment, with the only use of a fume-hood.

The purpose of this study was optimizing an already existing method for simultaneous DNA and RNA co-extraction [1] and apply it on adult unengorged and semi-engorged tick specimens using TRIzol^Ⓡ^ LS. In addition to the standard DNA isolation procedure following TRIzol^Ⓡ^ LS protocol, interphase/organic phases obtained during TRIzol^Ⓡ^ LS lysis were alternatively subjected to DNA purification using a commercial kit, with the intent of reducing the procedure time and obtaining a more purified nucleic acid.

As far as we know, DNA and RNA co-extraction has never been described in detail for ticks, in particular by using this reagent, and there are no specific reports on this topic. Moreover, in this protocol we tested the amplifiability of both isolated DNA and reverse-transcribed cDNA through a PCR approach.

This optimization grants that both RNA and DNA could be used for specific purposes, such as: (i) epidemiological analyses on the presence of different DNA- and RNA-based TBPs which otherwise would be impossible to perform on the same sample; (ii) inactivation of infectious agents during the first steps of sample handling aimed at molecular biology analyses; (iii) transcriptomics analyses to obtain information on the interactions between microorganisms within the tick, and between the tick and its microbiome; (iv) gene expression analyses on specific target genes.

Additionally, once the sample has been disrupted in TRIzol^Ⓡ^ LS (as well as classic TRIzol^Ⓡ^ Reagent), RNA degradation is prevented, overcoming the use of stabilizing solutions (as the sample can be directly preserved and processed once crushed into TRIzol^Ⓡ^ LS), and samples can be stored at + 4 °C overnight or at -20 °C for longer periods.

## Declaration of Competing Interest

The authors declare that they have no known competing financial interests or personal relationships that could have appeared to influence the work reported in this paper.

## Appendix Supplementary materials

Image, application 1
