# Supplementary material for: Protocol optimization for simultaneous DNA and RNA co-extraction from single hard tick specimens
Source: MethodsX. 2021 Mar 21;8:101315. doi: 10.1016/j.mex.2021.101315 (PMC8374261; doi:10.1016/j.mex.2021.101315)

**Figure 1** Electropherogram views of the analyzed RNA samples obtained with the TapeStation Analysis software.
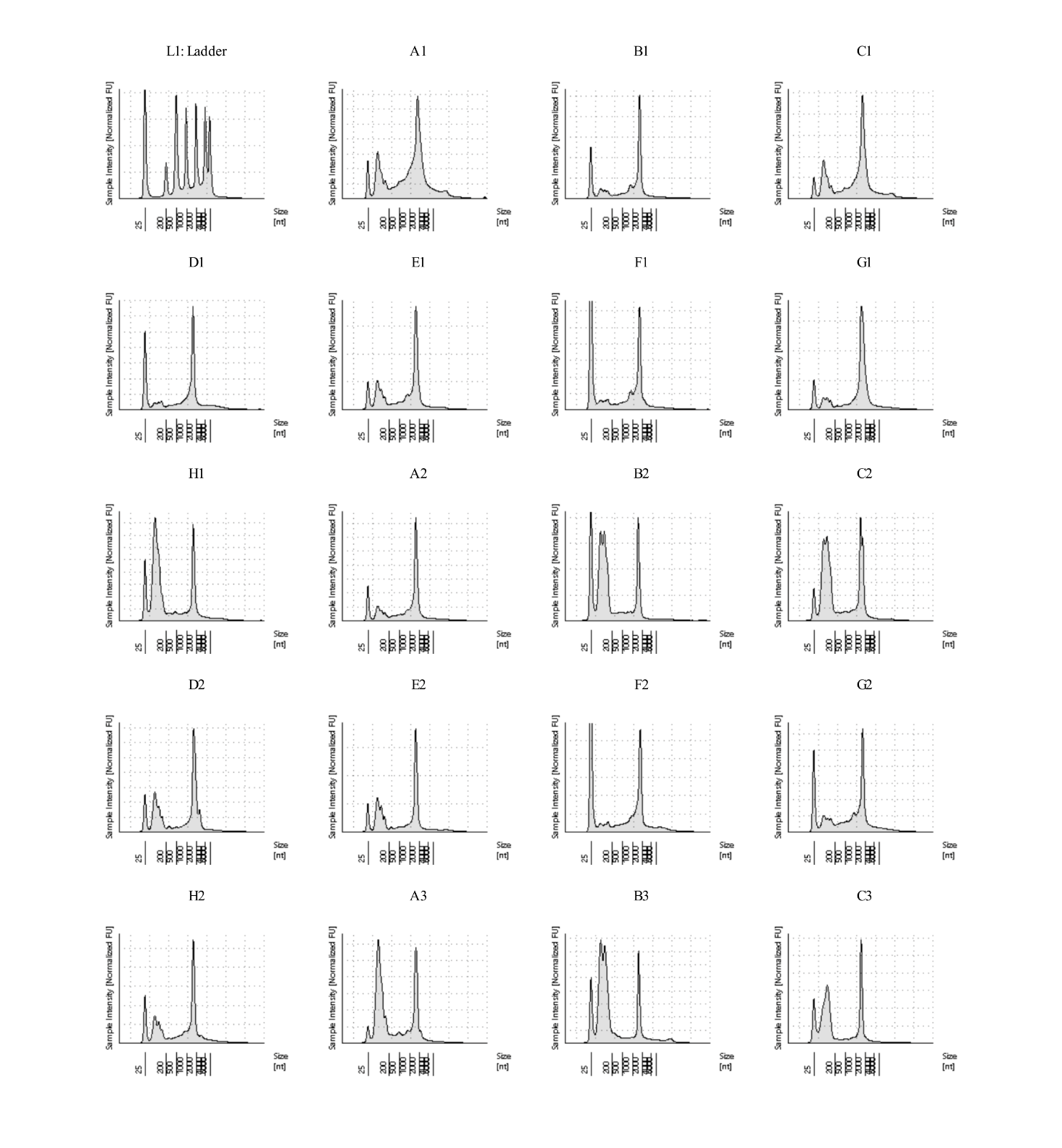

Supplement: Supplementary file 1 [file mmc1.docx]
